# Comparative metagenomic analysis reveals rhizosphere microbial community composition and functions help protect grapevines against salt stress

**DOI:** 10.3389/fmicb.2023.1102547

**Published:** 2023-02-20

**Authors:** Bo Wang, Xicheng Wang, Zhuangwei Wang, Kefeng Zhu, Weimin Wu

**Affiliations:** ^1^Institute of Pomology, Jiangsu Academy of Agricultural Sciences, Jiangsu Key Laboratory for Horticultural Crop Genetic Improvement, Nanjing City, Jiangsu Province, China; ^2^Department of Technology Commercialization, Jiangsu Academy of Agricultural Sciences, Nanjing City, Jiangsu Province, China; ^3^Huaian Herong Ecological Agriculture Co., Ltd, Huaian City, Jiangsu Province, China

**Keywords:** grapevine, rhizosphere, rootstock, microbial variation, metagenomics, sulfur metabolism

## Abstract

**Introduction:**

Soil salinization is a serious abiotic stress for grapevines. The rhizosphere microbiota of plants can help counter the negative effects caused by salt stress, but the distinction between rhizosphere microbes of salt-tolerant and salt-sensitive varieties remains unclear.

**Methods:**

This study employed metagenomic sequencing to explore the rhizosphere microbial community of grapevine rootstocks 101-14 (salt tolerant) and 5BB (salt sensitive) with or without salt stress.

**Results and Discussion:**

Compared to the control (treated with ddH_2_O), salt stress induced greater changes in the rhizosphere microbiota of 101-14 than in that of 5BB. The relative abundances of more plant growth-promoting bacteria, including Planctomycetes, Bacteroidetes, Verrucomicrobia, Cyanobacteria, Gemmatimonadetes, Chloroflexi, and Firmicutes, were increased in 101-14 under salt stress, whereas only the relative abundances of four phyla (Actinobacteria, Gemmatimonadetes, Chloroflexi, and Cyanobacteria) were increased in 5BB under salt stress while those of three phyla (Acidobacteria, Verrucomicrobia, and Firmicutes) were depleted. The differentially enriched functions (KEGG level 2) in 101-14 were mainly associated with pathways related to cell motility; folding, sorting, and degradation functions; glycan biosynthesis and metabolism; xenobiotics biodegradation and metabolism; and metabolism of cofactors and vitamins, whereas only the translation function was differentially enriched in 5BB. Under salt stress, the rhizosphere microbiota functions of 101-14 and 5BB differed greatly, especially pathways related to metabolism. Further analysis revealed that pathways associated with sulfur and glutathione metabolism as well as bacterial chemotaxis were uniquely enriched in 101-14 under salt stress and therefore might play vital roles in the mitigation of salt stress on grapevines. In addition, the abundance of various sulfur cycle-related genes, including genes involved in assimilatory sulfate reduction (*cysNC*, *cysQ*, *sat*, and *sir*), sulfur reduction (*fsr*), SOX systems (*soxB*), sulfur oxidation (*sqr*), organic sulfur transformation (*tpa*, *mdh*, *gdh*, and *betC*), increased significantly in 101-14 after treatment with NaCl; these genes might mitigate the harmful effects of salt on grapevine. In short, the study findings indicate that both the composition and functions of the rhizosphere microbial community contribute to the enhanced tolerance of some grapevines to salt stress.

## 1. Introduction

Soil salinization can be regarded as one of the most vital limiting factors of agricultural productivity and food security (Singh, 2021). High soil salinity often negatively affects plant growth and productivity by inducing oxidative stress, nutritional disorders, organ senescence, etc. (Yang and Guo, 2018). It is estimated that approximately 50% of the world’s agricultural soil will be salinized by the year of 2050 (Bharti et al., 2016). To address this issue, researchers have developed salt-tolerant plant varieties that can survive in high salinity soil using conventional breeding, transgenics, and CRISPR/Cas9 technology (Ashraf and Akram, 2009; İbrahimova et al., 2021; Tran et al., 2021). Meanwhile, some researchers have attempted to improve soil salinity *via* organic amendment (Rekaby et al., 2020; Kong et al., 2021). However, the above-mentioned methods are both labor and time-consuming.

An increasing number of studies have demonstrated that plant growth-promoting rhizobacteria (PGPR) can improve plant growth and productivity under salt stress (Numan et al., 2018). For example, colonizing the tomato rhizosphere with the maize rhizosphere bacteria *Bacillus* sp. MT7 improved the tolerance of tomatoes to 10% salt stress (Pathania et al., 2020). Additionally, combined application of rhizobia and *Pseudomonas* improved the growth performance of liquorice under salt stress (Egamberdieva et al., 2017). Moreover, fungi can regulate plant salt tolerance. Liu et al. (2015) reported that the indigenous arbuscular mycorrhizal fungal community promoted preferential plant uptake of K^+^ over Na^+^. There is no doubt that plant rhizosphere-associated microbes can help alleviate plant salt stress. However, the relationship between the salt tolerance of different plant genotypes with their rhizosphere microbiota remains unclear. A previous study reported that the rice *SST* (seedling salt tolerant) gene affected the assembly of the soil microbiome and its metabolites, thereby alleviating salt stress in rice (Lian et al., 2020). Thus, we hypothesized that the rhizosphere microbiota of genotypes with varying salt tolerances would affect plant performance to a different extent under salt stress.

Previous studies have reported a link between host genotype and rhizosphere bacteria (Briones et al., 2002). Aira et al. (2010) demonstrated that maize genotype strongly affected the rhizosphere microbial community, while Liechty et al. (2020) reported that rice genotype influenced methane-cycling microbes. Meanwhile, the rhizosphere microbial community can affect the abiotic stress tolerance of different plant genotypes. For example, the rhizosphere microbial communities of different soybean genotypes shaped distinct C decomposition and N transformation processes, which affected Al-stress tolerance (Li et al., 2020). Moreover, the water stress response of wheat was not only controlled by plant genotype, but was also associated with specific soil microbes (Azarbad et al., 2018). In addition, the rhizosphere microbial communities of different willow genotypes affected the Cd-stress response (Wang et al., 2021). To date, the impact of the rhizosphere microbiota on the salt stress tolerance of different plant genotypes has been ignored. However, plant roots directly face salinity challenges in the soil and acclimate the plant to salt stress. Therefore, research should focus on the rhizosphere microbial communities of different salt-tolerant genotypes in response to salt stress.

Grapes are one of the most cultivated fruits in the world, consumed both as fresh and processed products. As an important commercial fruit crop, the physiological and molecular mechanisms related to salt stress have been extensively investigated in grapevines, including the regulation of osmosis, ion responses, and reactive oxygen species (ROS), as well as mediation of transcriptional and noncoding RNAs (Galbiati et al., 2011; Ismail et al., 2014; Wang et al., 2016; Jin et al., 2020). Previous research has mostly focused on the impacts of salt stress on grapevine leaves and fruit (Lu et al., 2022; Liu, M. et al., 2022), while less attention has been paid to the root system. Several reports have adopted metagenomic strategies to characterize the grapevine microbiome under natural or biological stress conditions (Zhao et al., 2018; Azevedo-Silva et al., 2021). Therefore, the present work aimed to: (1) compare the rhizosphere microbiota of grapevine varieties with different salt tolerances; (2) clarify the regulatory mechanisms of rhizosphere microbes involved in enhancing grapevine adaptability to salt stress. Metagenomic analysis was performed on the rhizosphere microbiota of rootstocks from two grapevine varieties, including 101-14, which is known to have strong salt tolerance, and 5BB, which is known to have weak salt tolerance (Yuan et al., 2019).

## 2. Materials and methods

### 2.1. Plant materials, salt treatment, and rhizosphere soil sample collection

Strongly salt-resistant grapevine rootstock, 101-14 (*V. riparia* × *V. rupestis*), and weakly salt-resistant grapevine rootstock, 5BB (*V. berlandieri* × *V. riparia*), were cultivated under rain shelter in the vineyard of Jiangsu Academy of Agricultural Sciences, Nanjing, Jiangsu Province, China (32°02’ N, 118°52′ E). One cutting was planted per pot. One-year-old eight-leaf grapevine cuttings with consistent growth were used in the study. The experiment was performed in 2021 with average daytime and nighttime temperatures of 25°C and 10°C, separately, and 12 h light/12 h dark.

Potted grapevine cuttings (*n* = 18) were treated with 0.5 L 100 mM NaCl at 0, 48, 96, 144, and 192 h. An equal volume of ddH_2_O (0 mM NaCl) was used as the control treatment. The four treatment groups included: group G1, 101-14 treated with 100 mM NaCl (ST); group G2, 101-14 treated with 0 mM NaCl (SCK); group G3, 5BB treated with 100 mM NaCl (WT); and group G4, 5BB treated with 0 mM NaCl (WCK). Three grapevine cuttings were randomly selected and mixed together for a total of three replicates.

At 240 h (48 h after the last treatment), the rhizosphere soil was collected from the grapevine cuttings. The rhizosphere soil was sampled as previously described (Wang et al., 2015). The soil was passed through a 40-mesh sieve and kept at −70°C for DNA extraction.

### 2.2. DNA extraction and sequencing

Total DNA was extracted from the rhizosphere soil with a Soil DNA Kit (MP Biomedicals, Solon, OH, United States). DNA quality and concentration were then determined using 1% agarose gel and the Qubit dsDNA Assay Kit (Life Technologies, Carlsbad, CA, United States), respectively. Metagenomic sequencing of the rhizosphere soil total DNA was performed by Metware (Wuhan, China). The sequencing libraries were generated with the use of the NEBNext Ultra DNA Library Prep Kit for Illumina (New England Biolabs, Ipswich, MA, United States) following the instructions of the manufacturer. In brief, the DNA sample was fragmented by sonication to the size of 350 bp, and the DNA fragments were end-polished, A-tailed, and ligated with full-length adapters for Illumina sequencing with PCR amplification. After purification, the libraries were explored for size distribution using the Agilent 2,100 Bioanalyzer (Agilent Technologies, Santa Clara, CA, United States) and also quantified by real-time PCR. Index-coded samples were clustered using the cBot Cluster Generation System based on the guidance of the manufacturer (Illumina Inc., San Diego, CA, United States). Library preparations were sequenced on the Illumina NovaSeq platform (Illumina Inc.).

### 2.3. Pretreatment of sequencing results

Clean data were acquired by removing: (1) low quality reads (default quality threshold value <38) above a certain portion (default length of 40 bp); (2) reads with N base smaller than 10 bp; (3) reads above 15 bp overlapping with adapters. The clean data were aligned to the reference grape genome (*Vitis vinifera*, wine grape, BioProject: PRJEA18785) using Bowtie2.2.4. According to a previous description, the parameters were set (Karlsson et al., 2012, 2013).

### 2.4. Metagenome assembly, gene prediction, and abundance analysis

According to the previously described parameters, the clean data was assembled using MEGAHIT software (v1.0.4-beta; Qin et al., 2014). Mixed assembly was performed using SOAPdenovo (V2.04)/ MEGAHIT (v1.0.4-beta). In addition, fragments shorter than 500 bp in all scaftigs generated from both single and mixed assembly were filtered for performing the statistical analysis (Qin et al., 2014; Zeller et al., 2014).

Open reading frames (ORFs) were predicted from the assembled scaftigs (>500 bp) using MetaGeneMark (V2.10, http://topaz.gatech.edu/GeneMark/) software, and lengths shorter than 100 nt were filtered. CD-HIT (v4.5.8, http://www.bioinformatics.org/cd-hit) was adopted for omitting redundancies and obtaining the unique initial gene catalogue with the following parameter settings: -c 0.95, -M 0, -G 0 -aS 0.9, -g 1, -r 1, -d 0 (Zeller et al., 2014; Sunagawa et al., 2015).

The clean data of each sample were mapped to the initial gene catalogue by Bowtie2.2.4 software and the unigenes were acquired. The parameters were set in line with a previous description (Li et al., 2014). Based on the number of mapped reads and gene length, the abundance of each gene in each sample was calculated as follows: $G_{k}=\frac{r_{k}}{L_{k}}\cdot \frac{1}{\sum_{i=0}^{n}\frac{r_{i}}{L_{i}}}$, where *r* represents the number of reads mapped to genes and *L* represents the gene length. The summary statistics, core-pan gene analysis, and Venn diagram of gene counts were based on the abundance of each gene in each sample. Sulfur cycle genes were screened according to Yu et al. (2021).

### 2.5. Taxonomic predictions and functional annotations

The unigenes were aligned to sequences of the kingdoms Bacteria, Fungi, Archaea, and Viruses by DIAMOND software (Buchfink et al., 2015). Then, we extracted the sequences from the Non-Redundant database of NCBI (version 2018-01-02, https://www.ncbi.nlm.nih.gov/). For the final aligned results for each sequence, the alignment with the smallest e-value (Oh et al., 2014) was applied to the lowest common ancestor (LCA) algorithm for system classification using MEGAN (Huson et al., 2011) to verify the species annotation information. The number of genes and the abundance information for each sample in each taxonomy hierarchy were obtained. The abundance of a species in each sample was defined as the sum of the gene abundances for the species. The gene number for a species in a sample was the number of genes with a nonzero abundance. DIAMOND software (v0.9.9) with the settings –blastp and -e 1e-5 was used to align the unigenes to the KEGG (Kyoto Encyclopedia of Genes and Genomes) and eggNOG (Evolutionary Genealogy of Genes: Non-supervised Orthologous Groups, v4.5) databases. The relative abundance of each annotated functional hierarchy was determined. The functional annotation results and gene abundance data showed the number of genes associated with each taxonomic hierarchy was obtained for each sample. The relative abundance of each functional hierarchy was equal to the sum of relative abundances of genes annotated to the particular functional level. Based on the functional annotation results and gene abundance table, a gene number table for each sample in each taxonomy hierarchy was obtained. The gene number for a function in a sample was equal to the number of genes annotated to the function with a nonzero abundance.

### 2.6. Identification of core and representative features

The most abundant (>0.01%) and ubiquitous (prevalence = 1) taxa among all samples were selected as the core taxonomic and functional metagenomes. Screening was performed based on the “core_members” function in R version 4.2.1. Next, the differential abundance of taxonomic metagenomes was evaluated statistically based on linear discriminant analysis (LDA) effect size (LEfSe) with LEfSe software (Segata et al., 2011). The differential functional metagenomes were analyzed using two methods: (1) LEfSe analysis and (2) reporter score. The reporter scores were based on the Z-scores of individual KEGG orthologs (KOs). In addition, differentially enriched KEGG pathways were detected based on the Z-scores. Reporter scores >1.96 or <−1.96 (95% confidence), >2.58 or <−2.58 (99% confidence) were used as the detection threshold (Patil and Nielsen, 2005).

### 2.7. Analysis of microbial co-occurrence networks

The co-occurrence network was constructed as described by Khoiri et al. (2021). Briefly, the microbial abundance matrix was prepared and data were divided into four subsets (G1, G2, G3, and G4). Microbial species present at least 3 of 4 subsets with abundances of more than 11 were included in analyses. Next, 500 features of each data set were randomly selected for network construction to avoid contrast deviation caused by unequal numbers of features. Then, empirical sparse correlations for compositional data (SparCC) coefficients were calculated, and the network was constructed based on SparCC (Friedman and Alm, 2012) using FastSpar version 0.0.10 (Watts et al., 2019). Visualization of the network was performed using Gephi 0.9.6 software with the following conditions: |r| > 0.6 and *p* value < 0.05. Topological indices (i.e., node number, edge number, density, average degree, centrality, and modularity) were calculated. Within-module connectivity (Z_i_) and among-module connectivity (P_i_) were calculated for the module hubs (nodes with Z_i_ ≥ 2.5 and P_i_ < 0.62), connectors (nodes with Z_i_ < 2.5 and P_i_ ≥ 0.62), and network hubs (Z_i_ ≥ 2.5 and P_i_ ≥ 0.62; Sun et al., 2022).

### 2.8. Statistical analyses

Independent-samples *t*-tests were conducted using IBM SPSS 20.0. An unconstrained principal coordinate analysis (PCoA) based on Bray–Curtis distances was used to evaluate the differences in microbial composition and functions among samples.

## 3. Results

### 3.1. Metagenome sequencing results

In total, 520,524,840 raw sequence reads from 12 shotgun metagenome libraries were obtained, ranging from 39,656,770 to 45,766,780 reads per sample. After quality-filtering, >99% of reads were retained and the percentage of reads with a quality score ≥ 30 was >90%, indicating the data were sufficient for downstream analyses (Supplementary Table 1). Obviously, the rarefaction curves of core and pan genes tended to plateau, indicating that the samples covered the majority of the microbiota in the rhizosphere (Supplementary Figure 1). In addition, the average number of genes in each sample ranged from 1,019,332 to 1,375,565 in the four groups (Supplementary Figure 2).

### 3.2. Variations in rhizosphere microbiota after NaCl treatment

**Figure 1:**
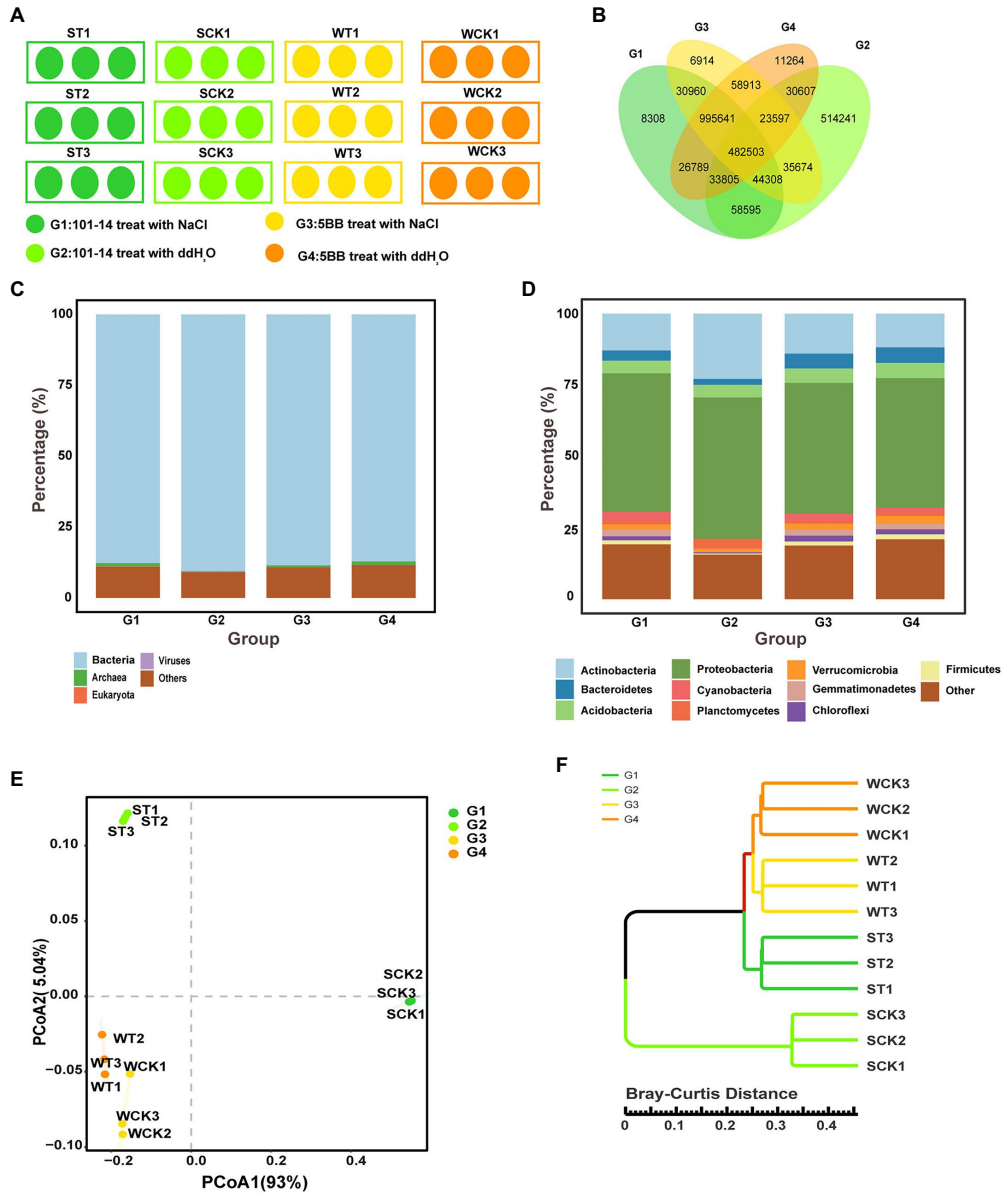
Rhizosphere soil microbiota of 101-14 and 5BB treated with NaCl and ddH_2_O. **(A)** Experimental design for pot cultivation study. **(B)** Overlapping OTUs enriched in different groups. Distribution of OTUs at the **(C)** kingdom and **(D)** phylum level in different groups. **(E)** Principle co-ordinates analysis based on Bray–Curtis distances of microbiota descriptions in different groups at the phylum level. **(F)** Cluster analysis based on Bray–Curtis distances at the genus level. G1, 101-14 treated with NaCl; G2, 101-14 treated with ddH_2_O; G3, 5BB treated with NaCl; G4, 5BB treated with ddH_2_O.

To explore the role of the rhizosphere microbiota in assisting grapevine adaptability to salt stress, the rootstocks of two different salt-tolerant grapevine varieties were subjected to salt stress or ddH_2_O (Figure 1A). The results indicated a significant overlap between the different groups. A total of 482,503 OTUs were enriched among the four groups, accounting for 28.70% in G1, 39.44% in G2, 28.75% in G3, and 29.01% in G4 (Figure 1B). The rhizosphere microbiota differed considerably between G1 and G2 at the kingdom and phylum levels, but the distribution of the rhizosphere microbiota was similar between G3 and G4 at the kingdom and phylum levels (Figures 1C,D). At the kingdom level, Bacteria was present in the four groups at higher relative abundance than Archaea, Eukaryota, and Viruses (Figure 1C). At the phylum level, Proteobacteria and Actinobacteria were discovered to be comparatively more abundant in the four groups than other phyla. The phyla Planctomycetes, Bacteroidetes, Verrucomicrobia, Cyanobacteria, Gemmatimonadetes, Chloroflexi, and Firmicutes were significantly more abundant in G1 than in G2, whereas the relative abundance of Actinobacteria was higher in G2 than in G1 (Figure 1D; Supplementary Table 2). Actinobacteria, Cyanobacteria, and Gemmatimonadetes were relatively more abundant in G3 than in G4, whereas Acidobacteria, Verrucomicrobia, Chloroflexi, and Firmicutes were relatively less abundant (Figure 1D; Supplementary Table 2).

Principal coordinate analysis (PCoA) on the basis of Bray–Curtis distances revealed that the rhizosphere microbiota of the four groups formed three distinct clusters alongside the first coordinate axis (Figure 1E), and the cluster analysis on the basis of Bray–Curtis distances at the genus level also exhibited the same clustering effect. G3 and G4 grouped into one cluster, which were separated from G1 and G2 (Figure 1F). These data suggested that the rhizosphere microbiota of 101-14 and 5BB differed considerably. In 101-14, the rhizosphere microbiota composition showed notable differences between the ddH_2_O- (G2) and NaCl-treated (G1) groups. In contrast, the rhizosphere microbiota composition of 5BB was similar between the ddH_2_O- (G4) and NaCl-treated (G3) groups.

### 3.3. NaCl treatment induces distinct changes in microbiota

**Figure 2:**
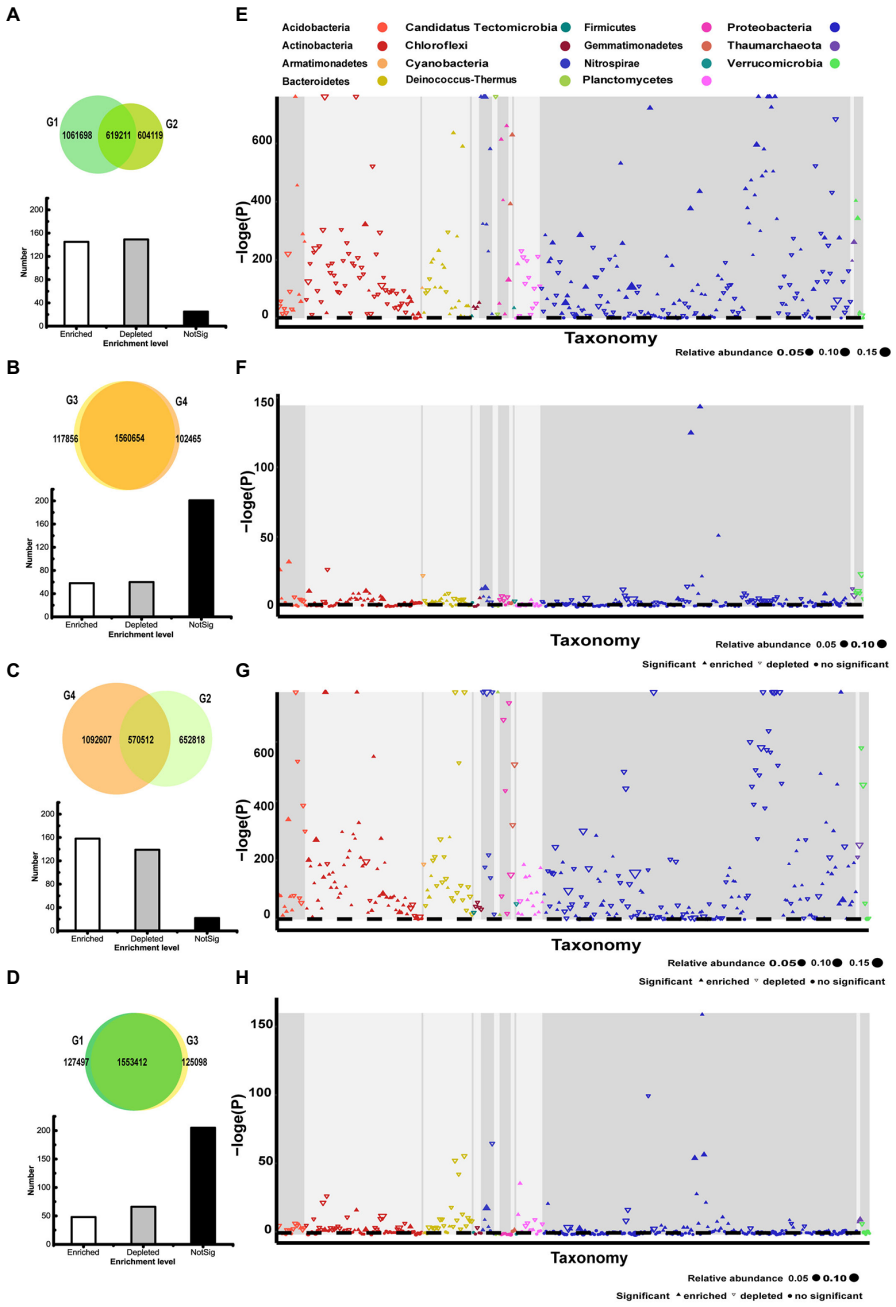
Taxonomic characteristics of differential OTUs between different groups. **(A)** Overlapping OTUs enriched in G1 and G2. **(B)** Overlapping OTUs enriched in G3 and G4. **(C)** Overlapping OTUs enriched in G2 and G4. **(D)** Overlapping OTUs enriched in G1 and G3. **(E)** Manhattan plot of OTUs enriched in G1 or G2. **(F)** Manhattan plot of OTUs enriched in G3 or G4. **(G)** Manhattan plot of OTUs enriched in G2 or G4. **(H)** Manhattan plot of OTUs enriched in G1 or G3. G1, 101-14 treated with NaCl; G2, 101-14 treated with ddH_2_O; G3, 5BB treated with NaCl; G4, 5BB treated with ddH_2_O.

Differences in the rhizosphere microbiota of 101-14 and 5BB under salt stress were explored at the OTU level. A total of 619,211 enriched OTUs (36.84% in G1, 50.62% in G2) overlapped in the ddH_2_O- and NaCl-treated groups of 101-14 (Figure 2A). In comparison, 1,560,654 enriched OTUs (92.98% in G3, 93.84% in G4) overlapped in the ddH_2_O- and NaCl-treated groups of 5BB (Figure 2B). The OTUs were arranged according to their taxonomy and enrichment level in Manhattan plots (Figures 2E,F; Supplementary Table 3). The results revealed no taxonomic alterations between the four groups at the phylum level. Proteobacteria, Actinobacteria, Bacteroidetes, Acidobacteria, and Planctomycetes were the main enriched phyla in all groups. However, significant alterations were detected at the genus level. In 101-14, 145 OTUs were enriched, 149 OTUs were depleted, and 25 OTUs were not significantly changed at the genus level under salt stress (G1 vs. G2, Figure 2A). In 5BB, 58 OTUs were enriched, 60 OTUs were depleted, and 201 OTUs were unchanged at the genus level under salt stress (G3 vs. G4, Figure 2B). The contrast in microbiota enrichment between the two grapevine varieties under salt stress was notable. Among Acidobacteria, 15 genera were detected and their enrichment differed between 101-14 and 5BB under salt stress. Among Actinobacteria, 63 genera were detected and only 5 genera were enriched to the same extent in 101-14 and 5BB under salt stress. Among Proteobacteria, 169 genera were detected and only 30 genera were enriched to the same extent in 101-14 and 5BB under salt stress (Supplementary Table 3E). The results revealed that the rhizosphere microbiota of 101-14 and 5BB had different responses to salt stress.

Subsequently, microbiota enrichment was compared among NaCl- and ddH_2_O-treated groups. A total of 570,512 enriched OTUs (46.64% in G2, 34.30% in G4) overlapped in 101-14 and 5BB after treatment with ddH_2_O (G4 vs. G2, Figure 2C). However, 1,553,412 enriched OTUs (92.41% in G1, 92.55% in G3) overlapped in 101-14 and 5BB after treatment with NaCl (G1 vs. G3, Figure 2D). Strikingly, a similar trend was observed confirming that the rhizosphere microbiota of 101-14 and 5BB behaved differently under salt stress (Supplementary Table 3F). The results indicated that microbiota enrichment differed between grapevine varieties and after NaCl treatment.

### 3.4. Changes in core and specific microbes after NaCl treatment

Core microbes were selected based on their relative abundance (>0.01%) and ubiquitousness (prevalence = 1) across all samples. A total of 81 bacteria were determined to be core species in the grapevine rhizosphere, accounting for 0.55% of total observed taxa (Supplementary Figure 3). Species within the phyla Proteobacteria, Actinobacteria, and Acidobacteria were predominant taxa. *Pseudolabrys* sp. Root 1,462, *Alphaproteobacteria* bacterium 62-8, *Alphaproteobacteria* bacterium 64-11, *Solirubrobacterales* bacterium 70-9, *Gammaproteobacteria* bacterium 13_2_20CM_66_19, and *Actinobacteria* bacterium 13_1_20CM_4_69_9 (Supplementary Figure 3) were among the top core species.

**Figure 3:**
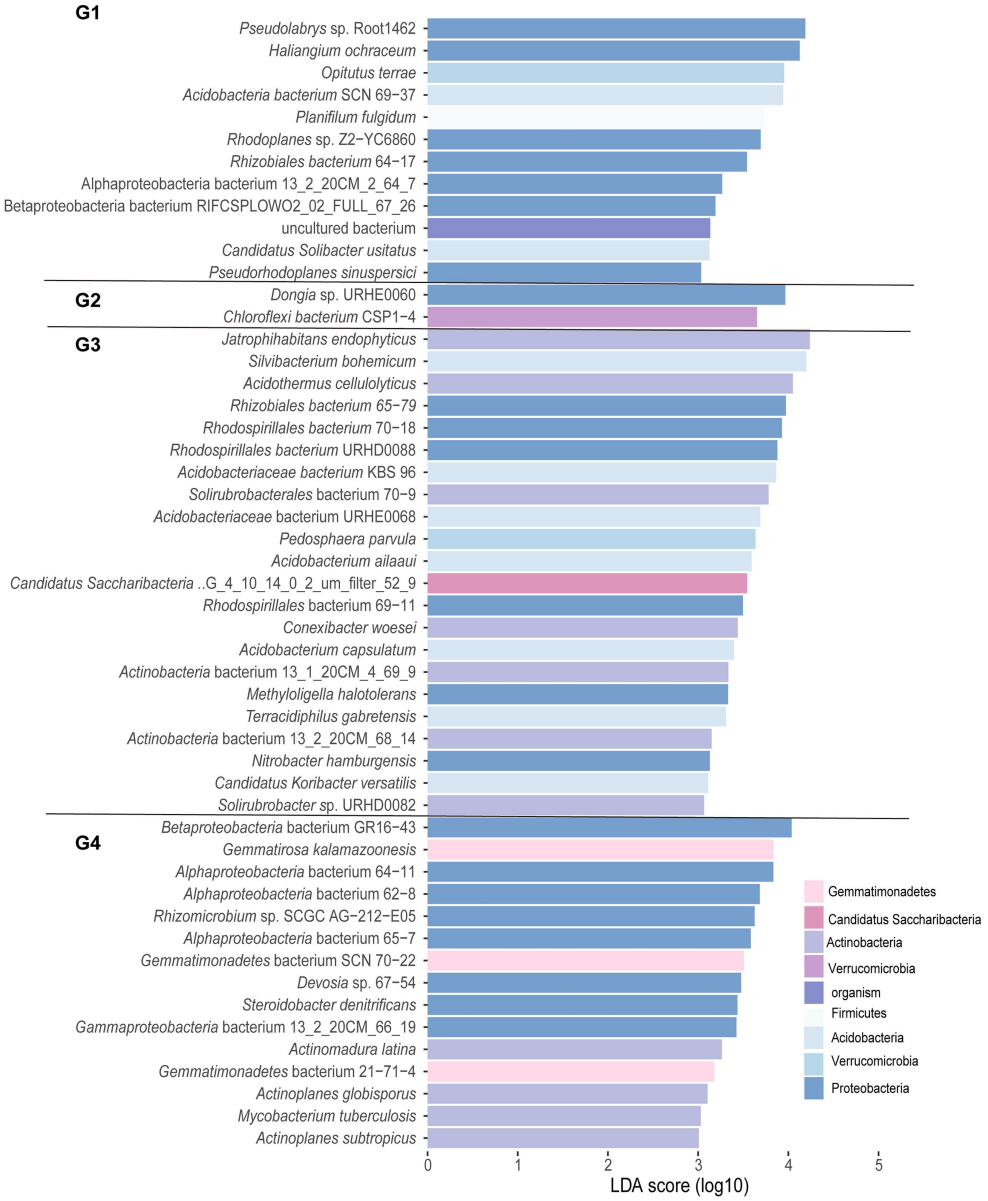
Histogram of linear discriminant analysis (LDA) analysis for different groups at the species level. Significance was determined *via* LDA effect size (LEfSe) analysis at *p* < 0.05 (Kruskal–Wallis test) and LDA score > 3. G1, 101-14 treated with NaCl; G2, 101-14 treated with ddH_2_O; G3, 5BB treated with NaCl; G4, 5BB treated with ddH_2_O.

In addition, representative core microbes of each group were determined by LEfSe analysis (LDA score > 3.0, *p* < 0.05). As shown in Figure 3, G1, G2, G3, and G4 had 15, 22, 2, and 12 enriched taxa, respectively. The unique species in the ddH_2_O-treated group of 5BB (G4) belonged to the phyla Proteobacteria, Verrucomicrobia, Acidobacteria, and Firmicutes. Treatment with NaCl decreased the number of unique species in 5BB (G3) to two, including *Dongia* sp. URHE0060 and *Chloroflexi* bacterium CSP1-4, which belonged to Proteobacteria and Chloroflexi, respectively. The ddH_2_O-treated group of 101-14 (G2) exhibited the highest number of enriched taxa with species belonging to the phyla Actinobacteria, Acidobacteria, Proteobacteria, Verrucomicrobia, Gemmatimonadetes, and Candidatus Saccharibacteria. However, treatment with NaCl reduced the number of unique species in 101-14 (G1), with species belonging to Proteobacteria, Gemmatimonadetes, and Actinobacteria. Furthermore, unique species belonging to Actinobacteria were only observed in the ddH_2_O- and NaCl-treated groups of 101-14 and not in those of 5BB.

### 3.5. Microbial co-occurrence networks

**Figure 4:**
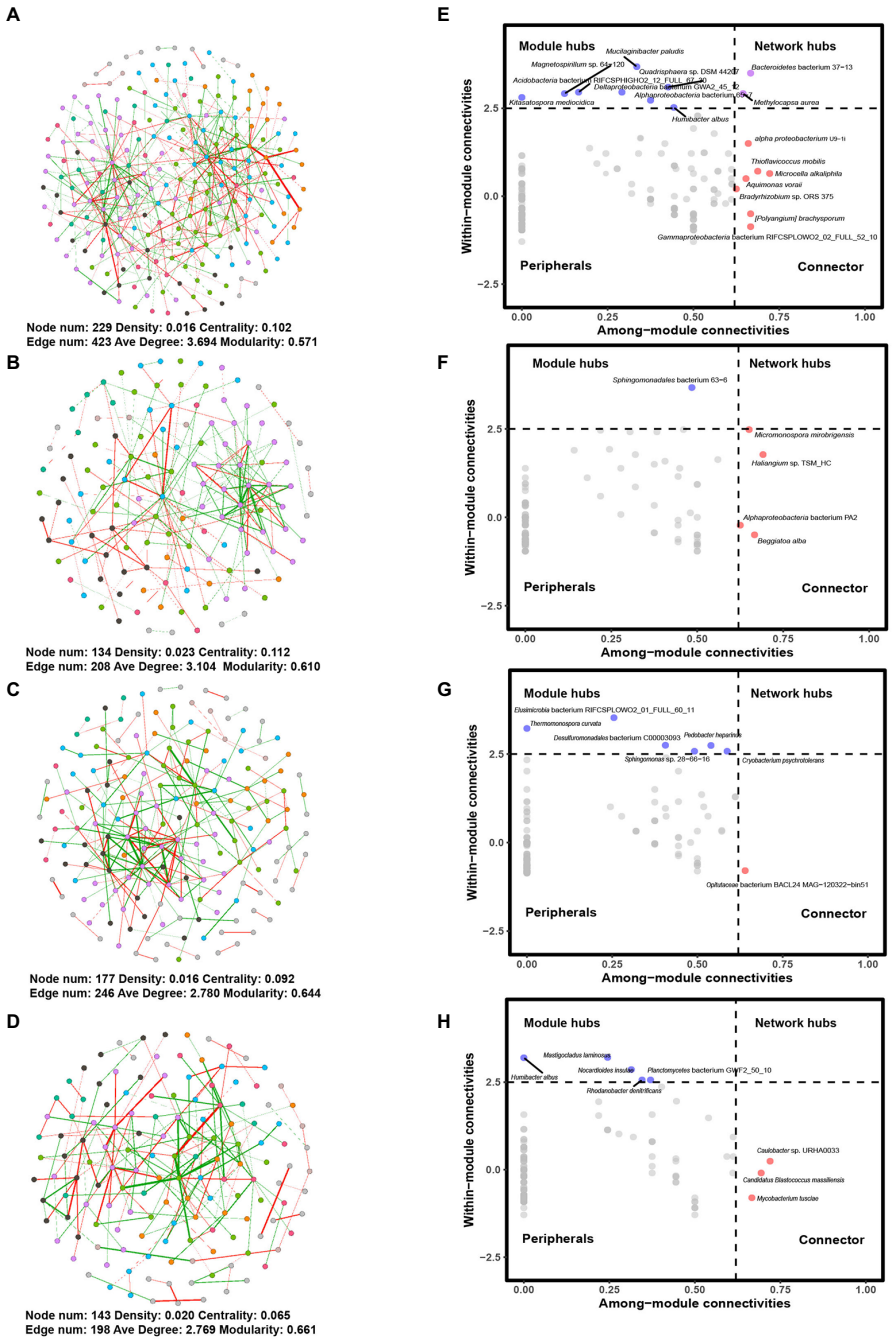
Networks and Z_i_-P_i_ plots for different groups. Networks for **(A)** G1, **(B)** G2, **(C)** G3, and **(D)** G4 and Z_i_-P_i_ plots for **(E)** G1, **(F)** G2, **(G)** G3, and **(H)** G4. The colors of nodes indicate network modules. Edges in red and green refer to positive and negative correlations, respectively. G1, 101-14 treated with NaCl; G2, 101-14 treated with ddH_2_O; G3, 5BB treated with NaCl; G4, 5BB treated with ddH_2_O.

In a co-occurrence network analysis, network connectivity was highest for 101-14 treated with NaCl (G1) (Figure 4). The total nodes and edges in G1 were 229 and 423, compared with 134 nodes and 208 edges in G2. The same trend was observed in G3 and G4. There were 177 nodes and 246 edges in G3 and 143 nodes and 198 edges in G4. In addition, the average degree increased after treatment with NaCl both in 101-14 and 5BB. However, the density decreased after treatment with NaCl in both 101-14 and 5BB. Centrality and modularity differed between 101-14 and 5BB. Network centrality and modularity decreased after treatment with NaCl in 101-14 but increased in 5BB. These results collectively indicated that NaCl treatment altered the microbial network, with different effects on salt-resistant and salt-sensitive rootstock.

Next, the roles of individual members were analyzed by a Z_i_-P_i_ plot. Based on the Z_i_ and P^i^ values, seven connectors (*Aquimonas voraii*, *Bradyrhizobium* sp. ORS 375, *Microcella alkaliphile*, *Thioflavicoccus mobilis*, *[Polyangium] brachysporum*, *alpha proteobacterium* U9-1i, and *Gammaproteobacteria* bacterium RIFCSPLOWO2_02_FULL_52_10), eight module hubs (*Humibacter albus*, *Kitasatospora mediocidica*, *Magnetospirillum* sp. 64-120, *Mucilaginibacter paludism*, *Quadrisphaera* sp. DSM 44207, *Acidobacteria* bacterium RIFCSPHIGHO2_12_FULL_67_30, *Alphaproteobacteria* bacterium 65-7, and *Deltaproteobacteria* bacterium GWA2_45_12) and two network hubs (*Methylocapsa aurea* and *Bacteroidetes* bacterium 37-13) were observed in 101-14 with NaCl treatment (Figure 4E), while four connectors (*Beggiatoa alba*, *Haliangium* sp. TSM_HC, *Micromonospora mirobrigensis*, and *Alphaproteobacteria* bacterium PA2) and one module hub (*Sphingomonadales* bacterium 63-6) were in detected in 101-14 (Figure 4F). In contrast, only one connector (*Opitutaceae* bacterium BACL24 MAG-120322-bin51) and six module hubs (*Cryobacterium psychrotolerans*, *Pedobacter heparinus*, *Sphingomonas* sp. 28-66-16, *Thermomonospora curvata*, *Desulfuromonadales* bacterium C00003093, and *Elusimicrobia* bacterium RIFCSPLOWO2_01_FULL_60_11) were detected in 5BB with salt treatment (Figure 4G) while there were three connectors (*Candidatus Blastococcus massiliensis*, *Caulobacter* sp. URHA0033, and *Mycobacterium tusciae*) and five module hubs (*Humibacter albus*, *Mastigocladus laminosus*, *Nocardioides insulae*, *Rhodanobacter denitrificans*, and *Planctomycetes* bacterium GWF2_50_10) in 5BB (Figure 4H). Overall, NaCl treatment resulted in greater network complexity in salt-resistant grapevine rootstock.

### 3.6. Functional composition of rhizosphere microbiota

A total of 6,683 KOs attributed to 397 KEGG pathways were identified among all samples. The gene functions covered six categories, with metabolism-associated pathways accounting for the greatest number of KOs (18.6–19.4%). Pathways in association with the metabolism of carbohydrates, amino acids, energy, cofactors, and vitamins were regarded as the top functional categories at KEGG level 2 (Supplementary Figures 4D,E). Further analysis revealed that purine metabolism (ko00230) was the predominant pathway at KEGG level 3, followed by ABC transporters (ko0210), two-component systems (ko02020), quorum sensing (ko02024), oxidative phosphorylation (ko00190), pyrimidine metabolism (ko00240), ribosomes (ko03010), pyruvate metabolism (ko00620), carbon fixation pathways in prokaryotes (ko00720), and glyoxylate and dicarboxylate metabolism (ko00630; Supplementary Figure 4F). PCoA based on Bray–Curtis distances demonstrated that G1 + G2 or G2 + G4 were separated from each other, but G3 + G4 or G1 + G3 clustered together (Supplementary Figures 4A–C).

Gene functions were further analyzed using the eggNOG database. At level 1, “function unknown” was more prevalent than other categories, followed by amino acid transport/metabolism and energy production/conversion functions (Supplementary Figure 5D). At level 2, (ABC) transporter, histidine kinase, and dehydrogenase functions were predominant (Supplementary Figure 5E). Additionally, ENOG410XNMH histidine kinase, serine threonine protein kinase (COG 0515), acriflavin resistance protein (COG0841), acyl-CoA dehydrogenase (COG1960), dehydrogenase (COG1012), two-component, sigma54 specific, transcriptional regulator, Fis family (COG2204), dehydrogenase reductase (COG1028), AMP-dependent synthetase/ligase (COG0318), aldehyde oxidase/xanthine dehydrogenase, molybdopterin binding (COG1529), and (ABC) transporter (COG1131) were identified (Supplementary Figure 5F). PCoA based on Bray–Curtis distances of function annotation abundance indicated that G3 + G4 or G1 + G3 clustered together at levels 1 and 2 and orthologous groups (OGs) (Supplementary Figures 5A–C). However, G2 was clearly separated from G4, and G1 and G2 were also separated. These trends were similar to those observed in the KEGG analysis (Supplementary Figures 5A–C).

### 3.7. Core and representative microbial functions after NaCl treatment

Core microbial functions across the four groups were determined by relative abundance (> 0.1%) and ubiquitousness (prevalence = 1). A total of 5 categories and 126 pathways were attributed to the core functions of the grapevine rhizosphere, and 89 metabolism-related pathways accounted for 70.6% of the total core microbial functions (Supplementary Figure 6). Pathways associated with the metabolism of amino acids (14), carbohydrates (15), and cofactors and vitamins (10), as well as to xenobiotics biodegradation and metabolism (11) were dominant (Supplementary Figure 6). No core microbial functions were attributed to KEGG pathways associated with human diseases.

**Figure 5:**
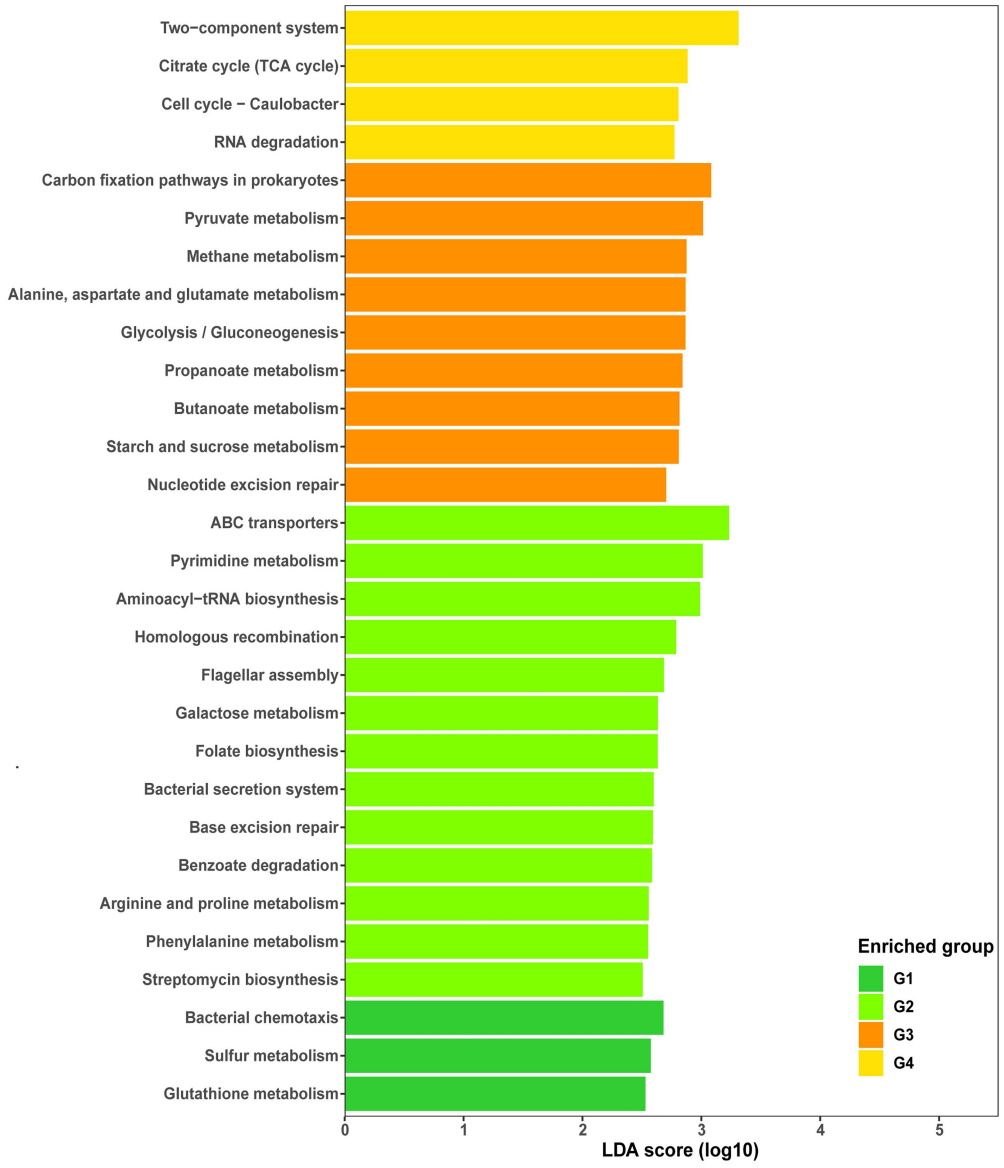
Histogram of linear discriminant analysis (LDA) of functional abundance in different groups using KEGG pathways. Significance was determined by LDA effect size (LEfSe) at *p* < 0.05 (Kruskal–Wallis test) and LDA score > 2.5. G1, 101-14 treated with NaCl; G2, 101-14 treated with ddH_2_O; G3, 5BB treated with NaCl; G4, 5BB treated with ddH_2_O.

Apart from the common core functions, representative core functions to each group were determined by LEfSe analysis (LDA score > 2.5, *p* < 0.05). Among KEGG functional pathways, those associated with bacterial chemotaxis, glutathione metabolism, and sulfur metabolism were uniquely enriched in G1, while 15 specific pathways were enriched in G2, including those associated with pyrimidine, arginine/ proline, galactose, phenylalanine, and biotin metabolism; benzoate degradation; and folate, streptomycin, and lysine biosynthesis (Figure 5). Similarly, eight pathways (88.89%) were differentially enriched in G3, including those associated with carbon fixation in prokaryotes; pyruvate, alanine/alanine/aspartate/glutamate, methane, propanoate, butanoate, and starch/sucrose metabolism; and glycolysis/gluconeogenesis. However, only four pathways, including those associated with two-component systems, the citrate cycle, cell cycle, and RNA degradation, were enriched in G4 (Figure 5).

**Figure 6:**
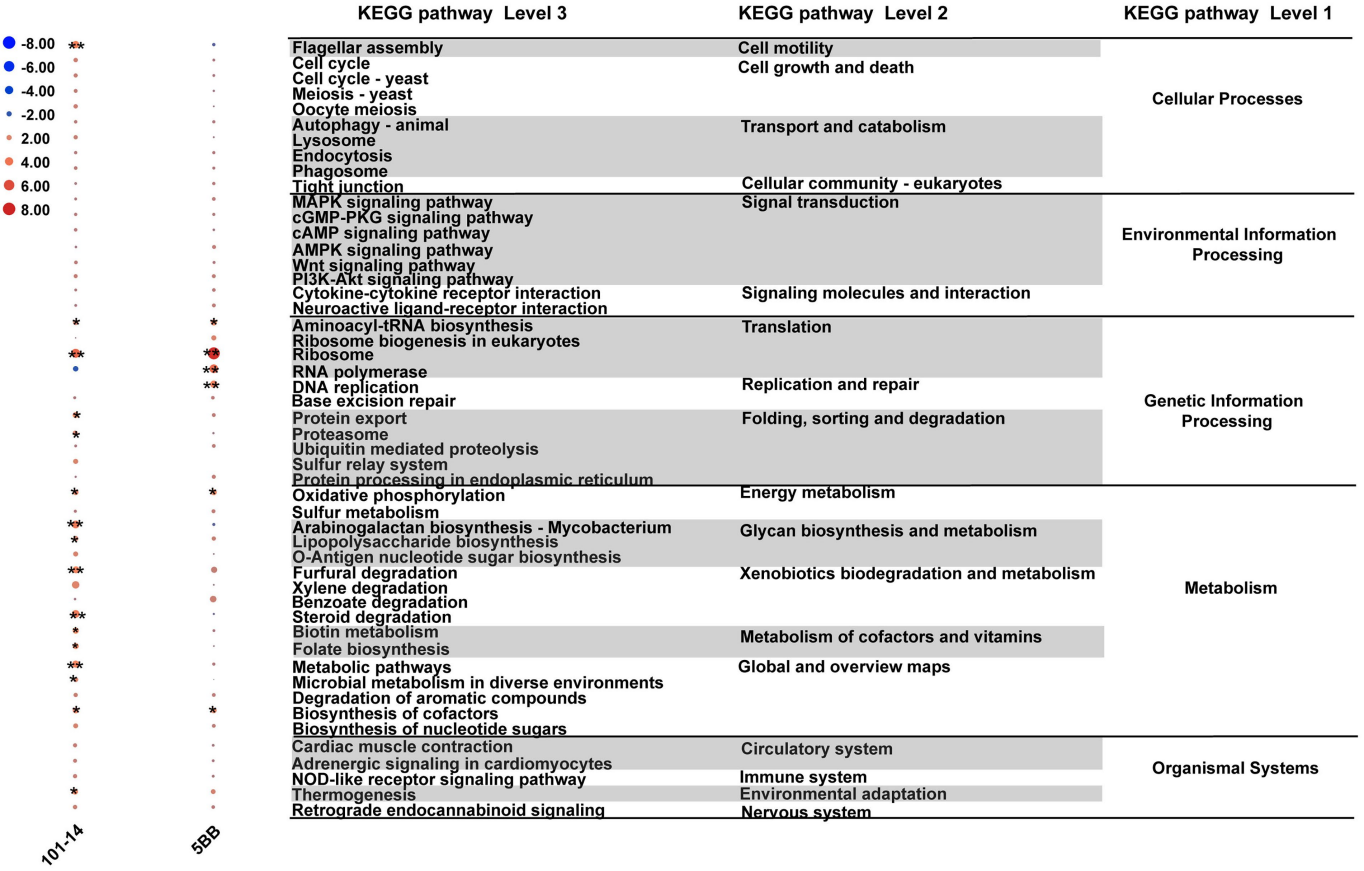
Heat map of top 50 KEGG pathways differentially enriched under salt stress in 101-14 and 5BB. Pathways are shown with a significant difference in reporter score. Blue, enriched in rhizosphere microbiota of both NaCl-treated varieties; red, enriched in rhizosphere microbiota of both ddH_2_O-treated varieties; green, enriched in rhizosphere microbiota of 101-14 treated with NaCl and ddH_2_O; orange, enriched in rhizosphere microbiota of 5BB in treated with NaCl and ddH_2_O. *denotes reporter score < −1.96, or > 1.96. **represent reporter score < −2.58, or >2.58. G1, 101-14 treated with NaCl; G2, 101-14 treated with ddH_2_O; G3, 5BB treated with NaCl; G4, 5BB treated with ddH_2_O.

**Figure 7:**
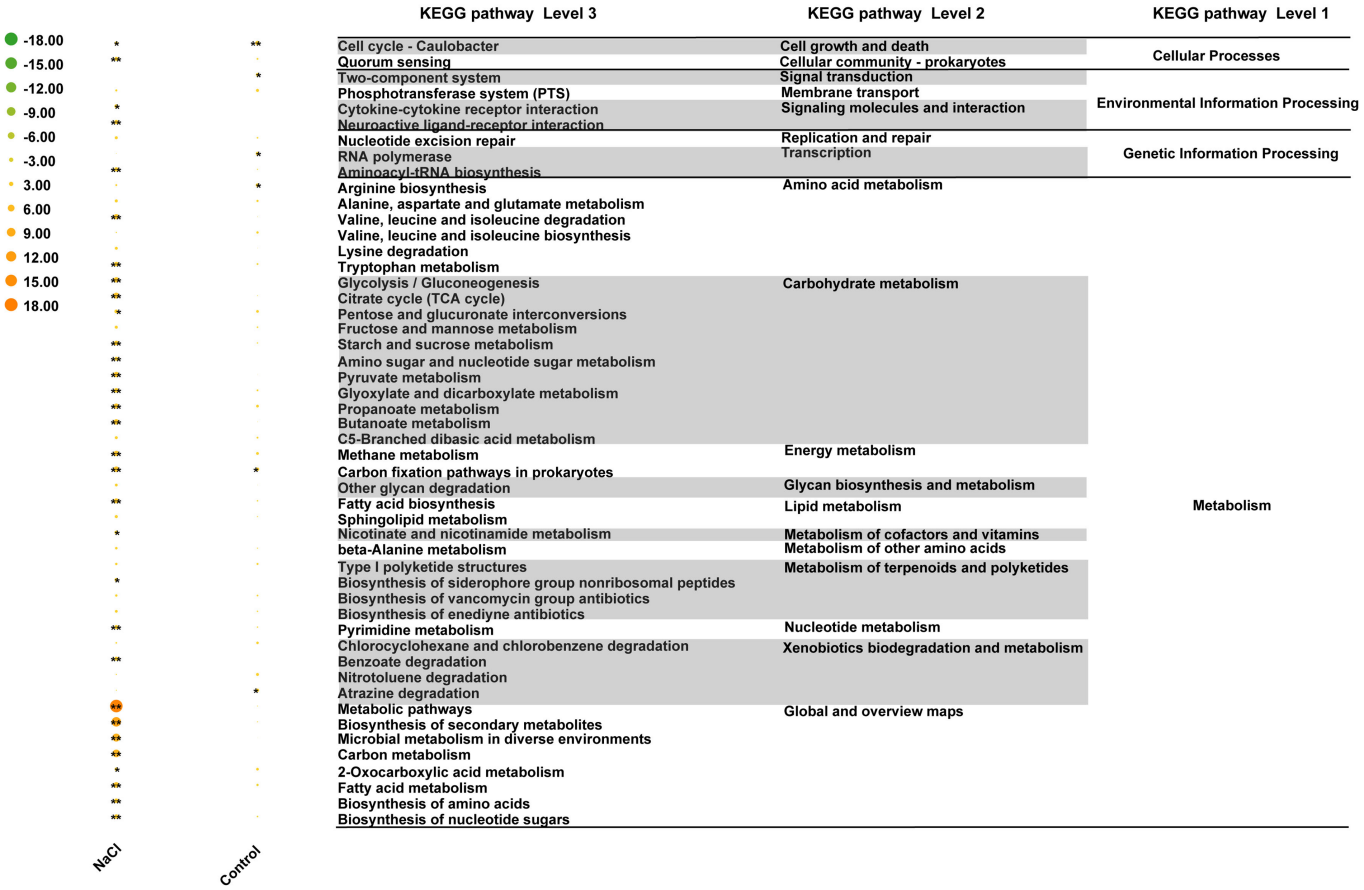
Heat map of top 50 KEGG pathways differentially enriched under salt stress in 101-14 and 5BB. Pathways are shown with a significant difference in reporter score. Green, enriched in rhizosphere microbiota of 101-14 treated with NaCl and ddH_2_O; orange, enriched in rhizosphere microbiota of 5BB in treated with NaCl and ddH_2_O. *denotes reporter score < −1.96, or > 1.96. **represent reporter score < −2.58, or >2.58. G1, 101-14 treated with NaCl; G2, 101-14 treated with ddH_2_O; G3, 5BB treated with NaCl; G4, 5BB treated with ddH_2_O.

Reporter score analysis based on KEGG pathways was also used to explore similarities and differences in the rhizosphere microbiota functions of the two grapevine varieties under salt stress. As shown in Figure 6, 17 and 1 discrepant pathways were enriched in the ddH_2_O- and NaCl-treated groups of 101-14, respectively. In contrast, only six discrepant pathways were enriched in the ddH_2_O-treated group of 5BB. Comparing the enriched pathways in 101-14 and 5BB, pathways associated with arabinogalactan biosynthesis-mycobacterium, biotin metabolism, flagellar assembly, folate biosynthesis, furfural degradation, lipopolysaccharide biosynthesis, metabolism, microbial metabolism in diverse environments, proteasomes, protein export, steroid degradation, thermogenesis, and xylene degradation were only depleted in 101-14 after NaCl treatment. In addition, enriched pathways in the ddH_2_O- and NaCl-treated groups were compared to determine whether the different functions were related to grapevine variety (Figure 7). Among the NaCl-treated groups, 27 and 4 discrepant pathways were enriched in 101-14 and 5BB, respectively. Among the ddH_2_O-treated groups, only six discrepant pathways were enriched in 101-14. Notably, the enriched pathways differed considerably between the NaCl- and ddH_2_O-treated groups of each grapevine variety.

### 3.8. Sulfur cycle genes expression pattern

**Figure 8:**
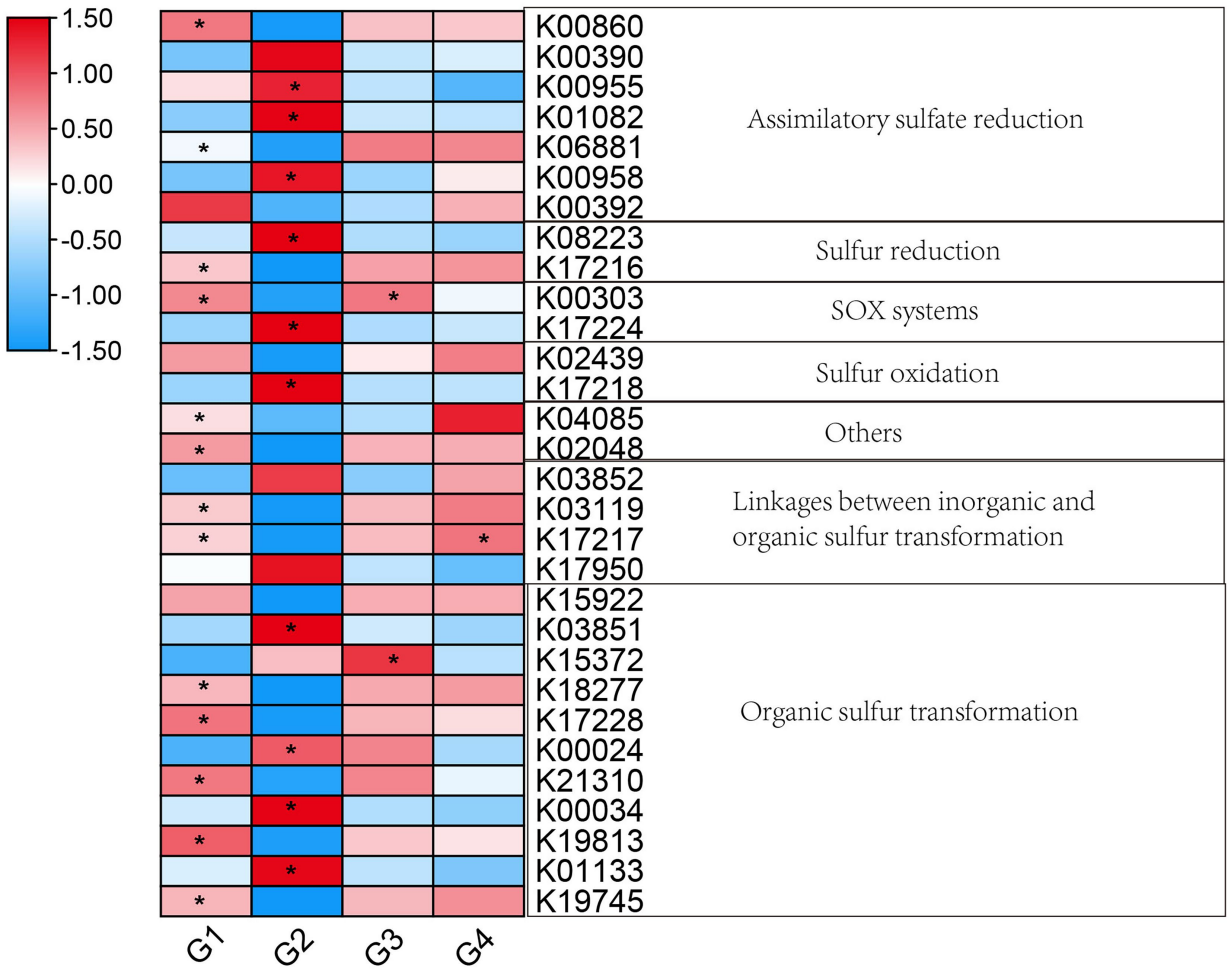
Heatmap of genes related to the sulfur cycle. Colors from blue to red indicate the log_2_ value (absolute abundance). *p < 0.05, independent-samples *t*-test was conducted for comparisons between G1 and G2, G3 and G4. G1, 101-14 treated with NaCl; G2, 101-14 treated with ddH_2_O; G3, 5BB treated with NaCl; G4, 5BB treated with ddH_2_O.

A total of 30 genes (KEGG Orthologues) related to the sulfur cycle were detected in the present study. These genes were involved in assimilatory sulfate reduction, sulfur reduction, SOX systems, linkages between inorganic and organic sulfur transformation, and organic sulfur transformation. The distribution of genes related to the sulfur cycle differed between 101-14 and 5BB. As shown in Figure 8, various genes showed significant differences between 101-14 with NaCl treatment and 101-14 with ddH_2_O treatment. Genes involved in assimilatory sulfate reduction (*cysNC*, *cysQ*, *sat*, and *sir*), sulfur reduction (*fsr*), SOX systems (*soxB*), sulfur oxidation (*sqr*), organic sulfur transformation (*tpa*, *mdh*, *gdh*, and *betC*) were enriched in 101-14 with NaCl treatment. Additional genes related to assimilatory sulfate reduction (*cysC* and *nrnA*), sulfur reduction (*mccA*), SOX system (*soxB*), other processed (*tusA* and *sbp*), linkages between inorganic and organic sulfur transformation (*tauD* and *mccB*), and organic sulfur transformation (*tmm*, *sfnG*, *mddA*, *gdh*, and *acuI*) were depleted after treatment with NaCl in 101-14. However, two genes, a SOX system gene (*soxB*) and organic sulfur transformation gene (*toa*), were depleted and one gene related to linkages between inorganic and organic sulfur transformation (*mccB*) was enriched in 5BB after treatment with NaCl (Figure 8). Overall, these results indicated that the abundance of sulfur cycle-related genes showed obvious differences in 101-14 between NaCl treatment and the control.

## 4. Discussion

The rhizosphere can be referred to as the narrow zone of soil regulated by root secretions, which forms the habitat of up to 10^11^ microbial cells/g root and over 30,000 prokaryotic species (Egamberdieva et al., 2008; Mendes et al., 2011). As previously noted, rhizosphere microbes exert a vital role in increasing plant tolerance to salt stress (Huang et al., 2014). However, rhizosphere microbes are varied and abundant. Therefore, a suitable approach is needed to uncover the specific roles of rhizosphere microbes in alleviating salt stress in plants. Shotgun metagenome sequencing employs whole genomic sequences to obtain more accurate taxonomic and functional categorization (Jovel et al., 2016). In the current work, shotgun metagenomic sequencing was adopted for investigating differences in the rhizosphere metagenomes between salt-sensitive (5BB) and salt-tolerant (101-14) grapevine varieties.

The taxonomic profiling results demonstrated that the rhizosphere microbiota of the two grapevine varieties shared the same 10 dominant phyla (Figure 1D). All of the identified phyla have been previously reported and are known to predominate grapevine rhizospheres (Zhang et al., 2019), as well as in those of peanut (Xu et al., 2020), pomegranate (Ravinath et al., 2022), barley (Bulgarelli et al., 2015), and *Arabidopsis thaliana* (Kielak et al., 2016). Nevertheless, the relative abundances of these phyla were distinct between 101-14 and 5BB (Figures 1E,F; Supplementary Table 2). Differences in rhizosphere microbiomes have been associated with different genotypes in some recent studies. For example, Chrysanthemum cultivars showed a strong selection for distinct rhizosphere microbes (Rotoni et al., 2022). Additionally, different rice (*Oryza sativa* L.) cultivars have been shown to recruit different rhizosphere microbes (Xiong et al., 2021). In the current work, the distinct effect of NaCl treatment was observed on the rhizosphere microbiota composition of the grapevine cultivars (Figures 1E,F, 2). These findings concurred with those of a previous study in which the relative abundances of beneficial bacteria, especially Cyanobacteria and Proteobacteria, were increased in salt-resistant peanut cultivars and decreased in salt-sensitive cultivar (Xu et al., 2022). In the present study, the relative abundances of seven major phyla that play positive roles in plant growth were increased in 101-14 under salt stress, but only the relative abundances of four phyla increased in 5BB under salt stress while the relative abundances of four phyla decreased.

The core microbiome is thought to be a critical component of the basic function of the holobiont, which shows a close relationship to the growth, health, and physiology of the host plant (Lemanceau et al., 2017; Zhou et al., 2020). According to Supplementary Figure 3, the core species belonging to nine phyla were the same as the dominant phyla in 101-14 and 5BB, except Cyanobacteria was not found among the core species (Figure 1D), and Candidatus Saccharibacteria was identified as a core species in 101-14. Candidatus Saccharibacteria was previously associated with partial denitrification in wastewater treatment and enriched in complex organic substrates (Remmas et al., 2017). *Pseudolabrys* sp. Root 1,462 was previously isolated from *A. thaliana*. Previous studies reported that *Pseudolabrys* has the ability to use nitrogen by converting NH4^+^-N fixation to microbial nitrogen, thus reducing nitrification and denitrification processes in soil (Li et al., 2022; Yu et al., 2022) Alphaproteobacteria is a dominant player in root-associated soil (Wu et al., 2009) and acts as a nitrogen fixer in coastal–saline soil ecosystems (Yousuf et al., 2014a). Gammaproteobacteria represent both nitrogen fixers and denitrifiers (Baskaran and Prabavathy, 2022). The presence of these core species suggested a significant contribution to the nitrogen cycle. Otherwise, some core species may play other vital roles in the response to soil salinity. For example, the relative abundance of *Solirubrobacterales* was significantly reduced by soil salinization (Yang et al., 2022). Gammaproteobacteria have been shown to participate in sulphur oxidation and inorganic carbon fixation in saline soil (Lenk et al., 2011; Yousuf et al., 2012, 2014b). Additionally, the presence of actinobacteria in the rhizosphere enabled plants to adapt to salt marsh environments (Rodriguez et al., 2008; Gong et al., 2018) by producing aminocyclopropane-1-carboxylate deaminase (ACCD) or exopolysaccharides (Vinothini et al., 2019; Mathew et al., 2020). ACCD can protect plants from salinity stress by decreasing the synthesis of the stress hormone ethylene *via* degradation of the precursor molecule, ACC, into α-ketobutyrate and ammonia (Mathew et al., 2020). Exopolysaccharides can enhance plant growth under salt stress through regulating stress-response genes (Vinothini et al., 2019). Indeed, a natural halotolerant actinobacterium *Glutamicibacter halophytocola* KLBMP 5180, isolated from a coastal halophyte, increased the salt tolerance of tomato, likely through the expression of genes associated with Na^+^/H^+^ exchange, K^+^ transport, etc. (Xue et al., 2017). Further, the results of the LDA analysis in the present study indicated that representative species belonging to the phylum Actinobacteria were only found in the rhizosphere of 101-14 (Figure 3). In general, the enrichment of PGPRs in the salt-tolerant grapevine rhizosphere might be the reason for the plant’s increased resistance to salt stress.

Co-occurrence networks were constructed in the present study to analyze the structure and complexity of the rhizosphere microbial communities (Xue et al., 2017) of grapevine varieties with different salt tolerances. Distinct keystone species were found in the four groups, indicating that both the plant cultivar and salt treatment caused shifts in the central taxa of the microbial community. These results aligned with those previously reported for other plants. For example, rice cultivars demonstrated different co-occurrence networks for their soil bacterial microbiomes (Xu et al., 2019). Plant cultivars are known to have a strong influence on rhizobacterial networks and keystone species (Jiang et al., 2017). Moreover, a previous study reported that water deficit changed the stability of plant rhizosphere networks (Bazany et al., 2022). In the current study, the microbial co-occurrence network of 101-14 was more complex than that of 5BB under salt stress. Zhao et al. (2022) found that high salt stress increased network connectivity in saline agricultural soils. However, Menendez-Serra et al. (2022) found that the microbial co-occurrence network was stable in ephemeral saline lakes under severe salinity fluctuations. Additionally, another study reported that the connectivity and complexity of the rhizosphere bacterial co-occurrence network were lowered in response to salt stress in *Nitraria tangutorum* (Pan et al., 2022). The conflicting results suggest that besides salt, plant root exudates should not be ignored in the construction of co-occurrence networks. Pan et al. (2022) reported that salt stress caused significant differences in the root exudates of *N. tangutorum*, especially organic acids, growth hormones, and sugars. In general, root exudate composition and quantity can change under abiotic stresses including drought (Vives-Peris et al., 2018) and salinization (Zhou et al., 2018). The present study did not analyze the root exudates of the two grapevine varieties, which should be involved in future studies.

In addition to the composition of the rhizosphere microbiota, this study also analyzed their functions. In a recent report, Zheng et al. (2021) suggested that microbial functions play key roles in enhancing plant resistance to salt stress and reported that genes related to cell motility, Na^+^ transport, and plant growth promotion were enriched in the rhizosphere of three different salt-tolerant plants. In the present study, metabolism was the predominant function in the grapevine rhizosphere no matter the cultivar or salt stress (Supplementary Figures 4D,E). The ABC transporter pathway was the core metabolism-related pathway in all groups (Supplementary Figure 6), as well as a uniquely enriched pathway in 101-14 (Figure 5). ABC transporters constitute transmembrane proteins and membrane-associated ATPases that help transfer carbohydrates, amino acids, inorganic ions, proteins, etc. (Finkenwirth and Eitinger, 2019). ABC transporter proteins are crucial for bacterial adaption to salt stress due to reduced molecule absorption, energy consumption, and regulation of osmotic pressure under high salinity conditions (Liu, J. T. et al., 2022). Further, a previous study found that ABC transporter genes were related to the bacterial response to osmotic stress, containing one specific locus that could mediate betaine accumulation to eliminate osmotic stress (Guo et al., 2022).

The functional analysis of the rhizosphere microbiota of 101-14 in response to salt stress indicated that sulfur and glutathione metabolism were uniquely enriched in 101-14 under salt stress. Plant growth processes can be significantly influenced by salt stress, especially photosynthesis. Photosynthesis is regulated by the availability of sulfur, which can be lowered by the harmful impacts of salt-induced oxidative stress (Nazar et al., 2015; Chan et al., 2019). Coordination of sulfur metabolism-related pathways between microorganisms and host plants is necessary to achieve salt stress tolerance in *Arabidopsis*. *Enterobacter* sp. SA187 was shown to promote sulfur metabolism in *Arabidopsis*, thereby increasing glutathione levels to mitigate ROS-induced damage (Andres-Barrao et al., 2021). Glutathione, a sulfur derivate, enhanced the antioxidant defense by maintaining a reduced cellular-redox level and scavenging supernumerary ROS under salt stress (Ruiz and Blumwald, 2002; Gill and Tuteja, 2010; Hasanuzzaman et al., 2018). Further studies have demonstrated that sulfur supplementation can improve the photosynthetic efficiency of plants under salt stress by increasing the production of glutathione (Fatma et al., 2014; Nazar et al., 2015). Sulfur cycle genes could be regulated by salinity. The abundances of some genes related to dissimilatory sulfur reduction and oxidation, the link between inorganic and organic sulfur transformation, and sulfur disproportionation and reduction decreased with increasing salinity, while genes involved in assimilatory sulfate reduction and sulfur oxidation tended to increase (Liu, Q. et al., 2022). In the present study, some genes related to the sulfur cycle increased in 101-14 after treatment with NaCl. For instance, *cysNC* encodes an enzyme that participates in converting sulfate into phosphoadenosine 5′-phosphosulfate (PAPS), *sat* can activate sulfate into adenosine 5′-phosphosulfate (APS), and sir is responsible for the reduction of sulfite to sulfide (Yu et al., 2021). Additionally, *fsr* is a coenzyme F420-dependent sulfite reductase gene (Susanti and Mukhopadhyay, 2012). The *tpa* gene is related to the conversion of C2 sulfonate (taurine, isethionate) into sulfoacetaldehyde (Durham et al., 2019; Landa et al., 2019). In this study indicated that genes involved in the sulfur cycle showed acute responses to salt stress in 101-14, and these genes might help mitigate the negative effect of salt on grapevine. In addition, bacterial chemotaxis was uniquely enriched in 101-14 under salt stress. Previous studies have shown that chemotaxis is a significant microbial function in which bacteria respond to (i.e., move towards or away from) chemotactic signals like root exudates or microbial metabolites (Neal et al., 2012; Lu et al., 2016; Sun et al., 2021). Chemotaxis towards root exudates is thought to be the first step of bacterial colonization (Sood, 2003). Some components of root exudates reportedly have a direct relationship with the chemotaxis reaction (Zhang et al., 2014). Notably, variations in root exudate composition under salt stress have also been demonstrated. Vives-Peris et al. (2018) reported that the proline and salicylate content of root exudates from a salt-tolerant genotype of *Citrus macrophylla* was higher than that from a salt-sensitive genotype. Bacterial growth was greater for the root exudate obtained from the salt-tolerant genotype than for the salt-sensitive genotype. These results indicated that bacterial chemotaxis is helpful to establish symbiosis with plant roots by the recruitment and colonization of microbes to salt-resistant rootstock rhizosphere. Taken together, the regulation of pathways associated with sulfur and glutathione metabolism and bacterial chemotaxis might be key mechanisms used by rhizosphere microbes in order to mitigate the harmful effects of salt stress on grapevines.

Taken together, our results clearly indicated that rootstock rhizosphere diversity and biological function, especially sulfur metabolism, contribute to relieving the harmful impacts of salt stress on grapevines. This work revealed great potential for further studies using microbiological or metabolic methods on salt-resistant cultivation technologies. Moreover, the results showed a correlation between the plant genotype and the influence of microbes on salt tolerance, and thus, the association between the soil microbiome and plant genotype should be further explored.

## 5. Conclusion

In this study, two grapevine rootstock genotypes showed significantly different salt tolerance responses. There was a more acute response and greater network complexity in the microbial community in the salt-tolerant vines, while in the salt-sensitive, a staler response was observed. When subjected to salt stress, the rhizosphere associated with the salt-tolerant vines exhibited a greater number and richness of growth-promoting bacteria than the salt-sensitive group. In addition, the abundance of genes related to the sulfur cycle increase significantly in salt-tolerant vines after treatment with NaCl. The microbial metabolism also showed more significant activity in salt-tolerant rootstocks. Particularly sulfur metabolism, glutathione metabolism, and bacterial chemotaxis pathways might have critical functions in enhancing grapevine tolerance to salt stress. In general, the obtained findings indicated that the effect of microbe-mediated tolerance to salt stress in grapevines is correlated to the grapevine genotype.

## Data availability statement

The datasets for this study can be found in the NCBI SRA [PRJNA900419; http://www.ncbi.nlm.nih.gov/sra/PRJNA900419].

## Author contributions

BW and WW designed the research. BW performed experiments and wrote the first manuscript. XW and KZ managed the field experiment. BW, XW, ZW, and KZ contributed to the evaluate the data and interpretation of the data. WW, ZW, and XW assisted with editing the paper. All authors contributed to the article and approved the submitted version.

## Funding

This research was supported by Jiangsu Province Breeding Project of Agricultural Leading New Varieties (PZCZ201722), China Agriculture Research System of MOF and MARA and Science and Technology Special Project for Subei of Jiangsu Province (XZ-SZ202101).

## Conflict of interest

KZ was affiliated with Huaian Herong Ecological Agriculture Co., Ltd.

The remaining authors declare that the research was conducted in the absence of any commercial or financial relationships that could be construed as a potential conflict of interest.

## Publisher’s note

All claims expressed in this article are solely those of the authors and do not necessarily represent those of their affiliated organizations, or those of the publisher, the editors and the reviewers. Any product that may be evaluated in this article, or claim that may be made by its manufacturer, is not guaranteed or endorsed by the publisher.

## Supplementary material

Supplementary Figure 1 Dilution curves of bacterial pan and core genes in the rhizosphere microbiota. **(A)** Core gene dilution curve. **(B)** Pan-gene dilution curve.

Supplementary Figure 2 Gene boxplot between different groups. G1, 101-14 treated with NaCl; G2, 101-14 treated with ddH_2_O; G3, 5BB treated with NaCl; G4, 5BB treated with ddH_2_O.

Supplementary Figure 3 Core microbial species among all samples. G1, 101-14 treated with NaCl; G2, 101-14 treated with ddH_2_O; G3, 5BB treated with NaCl; G4, 5BB treated with ddH_2_O.

Supplementary Figure 4 KEGG functions among all samples. Principle co-ordinates analysis based on Bray–Curtis distances of microbiota descriptions in different groups at **(A)** level 1, **(B)** level 2, and **(C)** level 3. Relative abundances at **(D)** level 1, **(E)** level 2, and **(F)** level 3. G1, 101-14 treated with NaCl; G2, 101-14 treated with ddH_2_O; G3, 5BB treated with NaCl; G4, 5BB treated with ddH_2_O.

Supplementary Figure 5 Functions identified using the eggNOG database among all samples. Principle co-ordinates analysis based on Bray–Curtis distances of microbiota descriptions in different groups at **(A)** level 1, **(B)** level 2, and **(C)** level 3. Relative abundances at **(D)** level 1, **(E)** level 2, and **(F)** level 3. G1, 101-14 treated with NaCl; G2, 101-14 treated with ddH_2_O; G3, 5BB treated with NaCl; G4, 5BB treated with ddH_2_O.

Supplementary Figure 6 Core microbial functions at KEGG level 3 among all samples. G1, 101-14 treated with NaCl; G2, 101-14 treated with ddH_2_O; G3, 5BB treated with NaCl; G4, 5BB treated with ddH_2_O.
