# Supplementary figures and images for: Comparative metagenomic analysis reveals rhizosphere microbial community composition and functions help protect grapevines against salt stress
Source: Front Microbiol. 2023 Feb 20;14:1102547. doi: 10.3389/fmicb.2023.1102547 (PMC9987714; doi:10.3389/fmicb.2023.1102547)

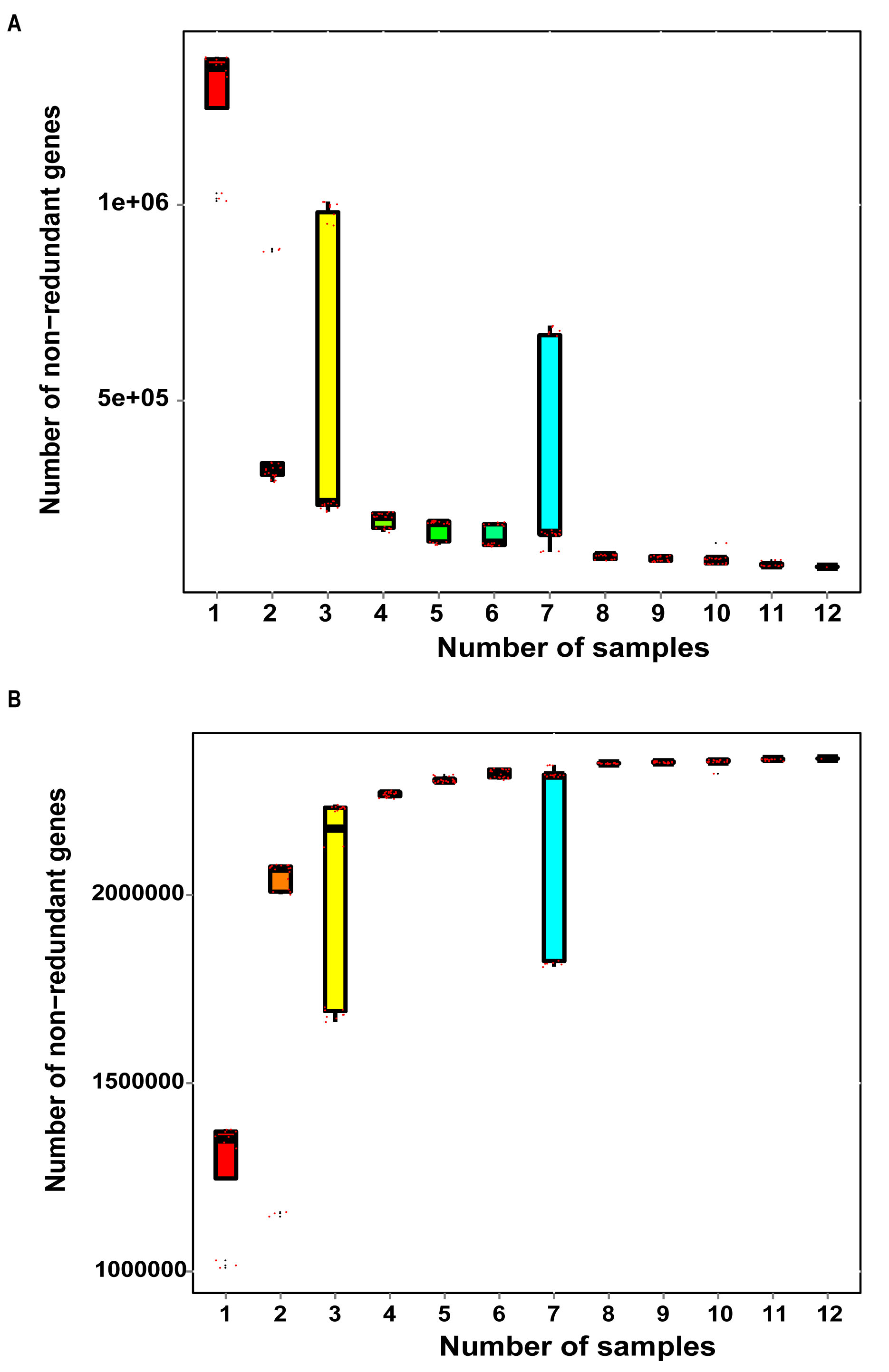

Supplement: Supplementary Figure 1 — Dilution curves of bacterial pan and core genes in the rhizosphere microbiota. (A) Core gene dilution curve. (B) Pan-gene dilution curve. [file Image_1.JPEG]

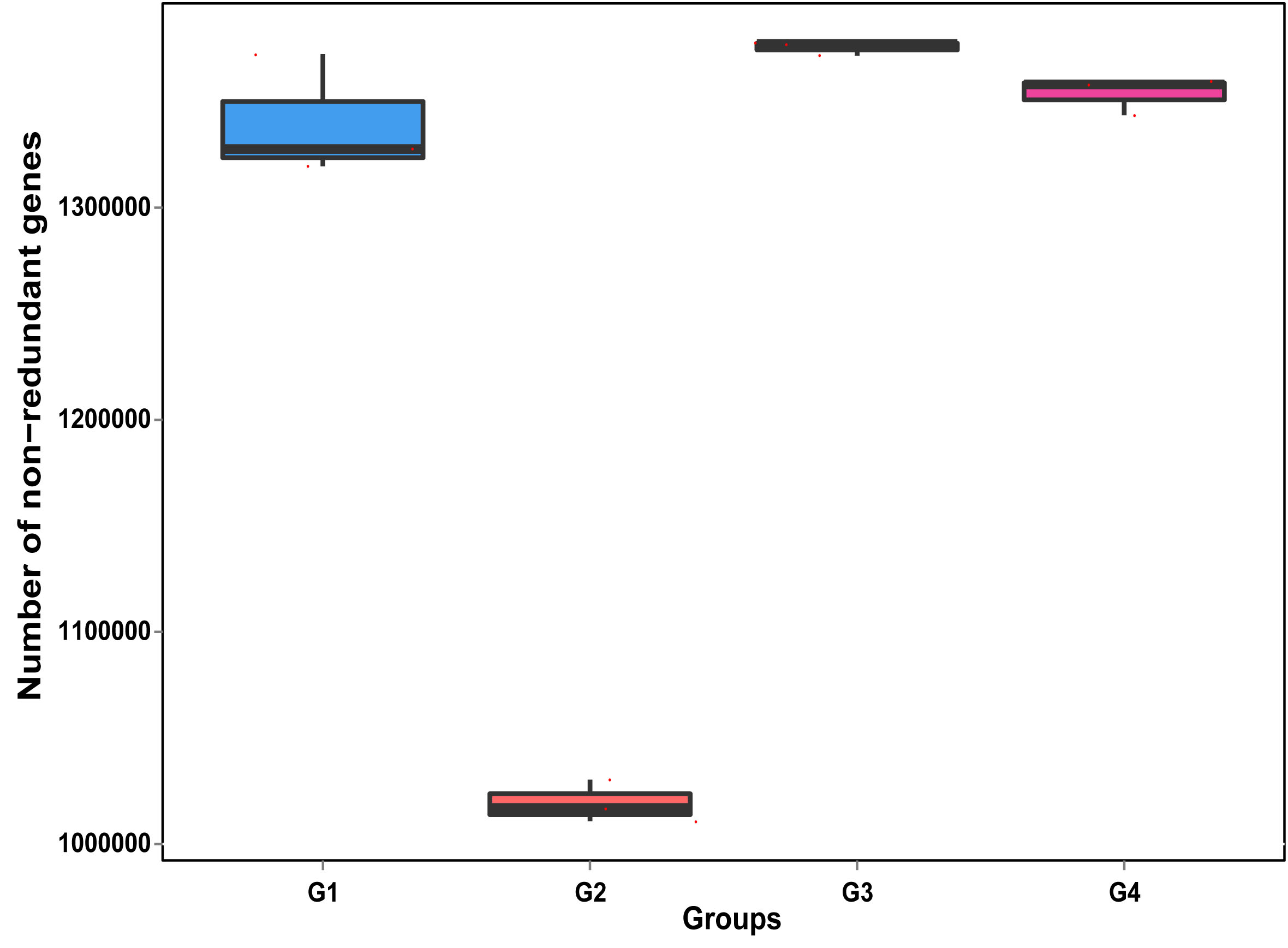

Supplement: Supplementary Figure 2 — Gene boxplot between different groups. G1, 101-14 treated with NaCl; G2, 101-14 treated with ddH2O; G3, 5BB treated with NaCl; G4, 5BB treated with ddH2O. [file Image_2.JPEG]

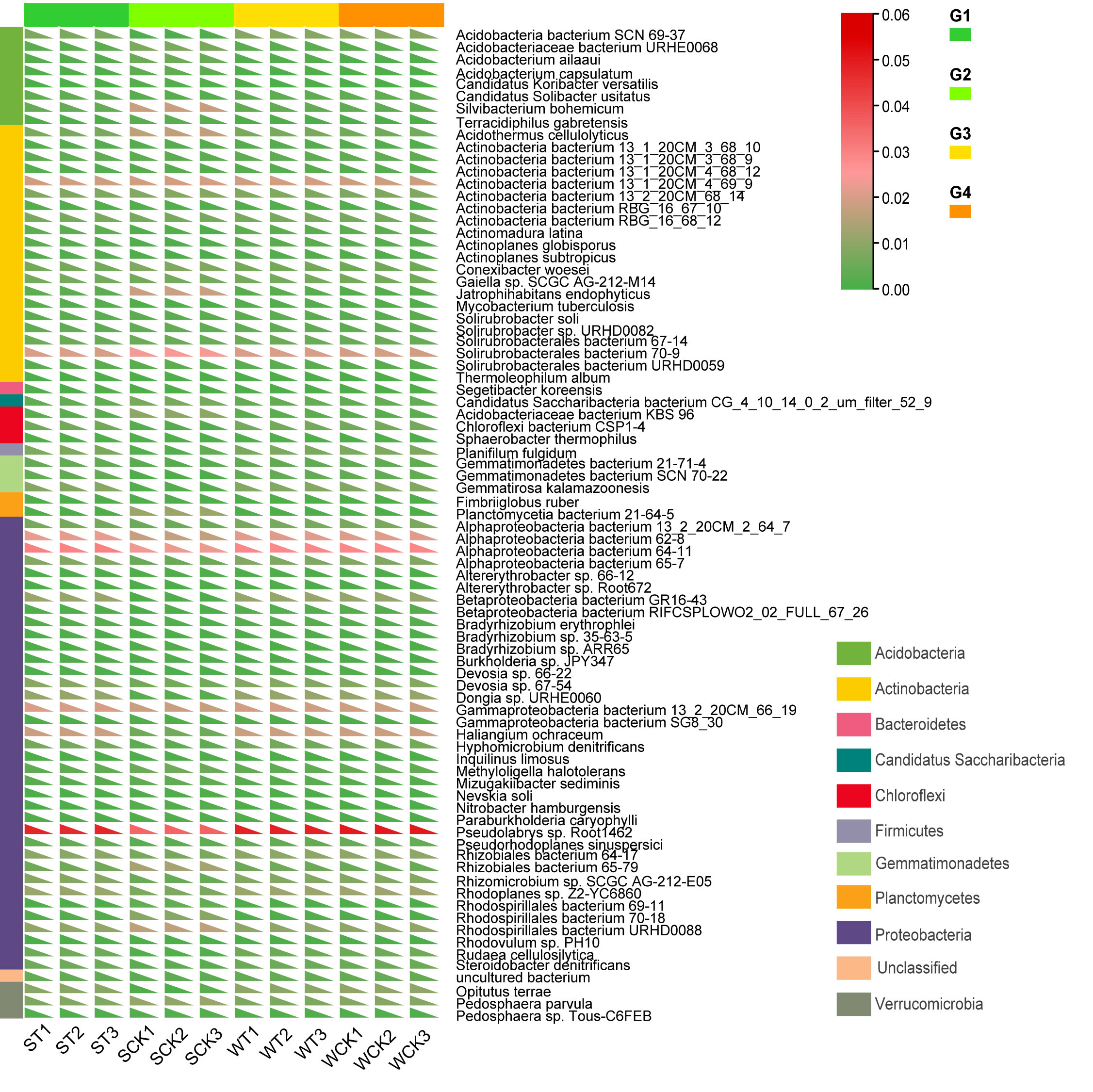

Supplement: Supplementary Figure 3 — Core microbial species among all samples. G1, 101-14 treated with NaCl; G2, 101-14 treated with ddH2O; G3, 5BB treated with NaCl; G4, 5BB treated with ddH2O. [file Image_3.JPEG]

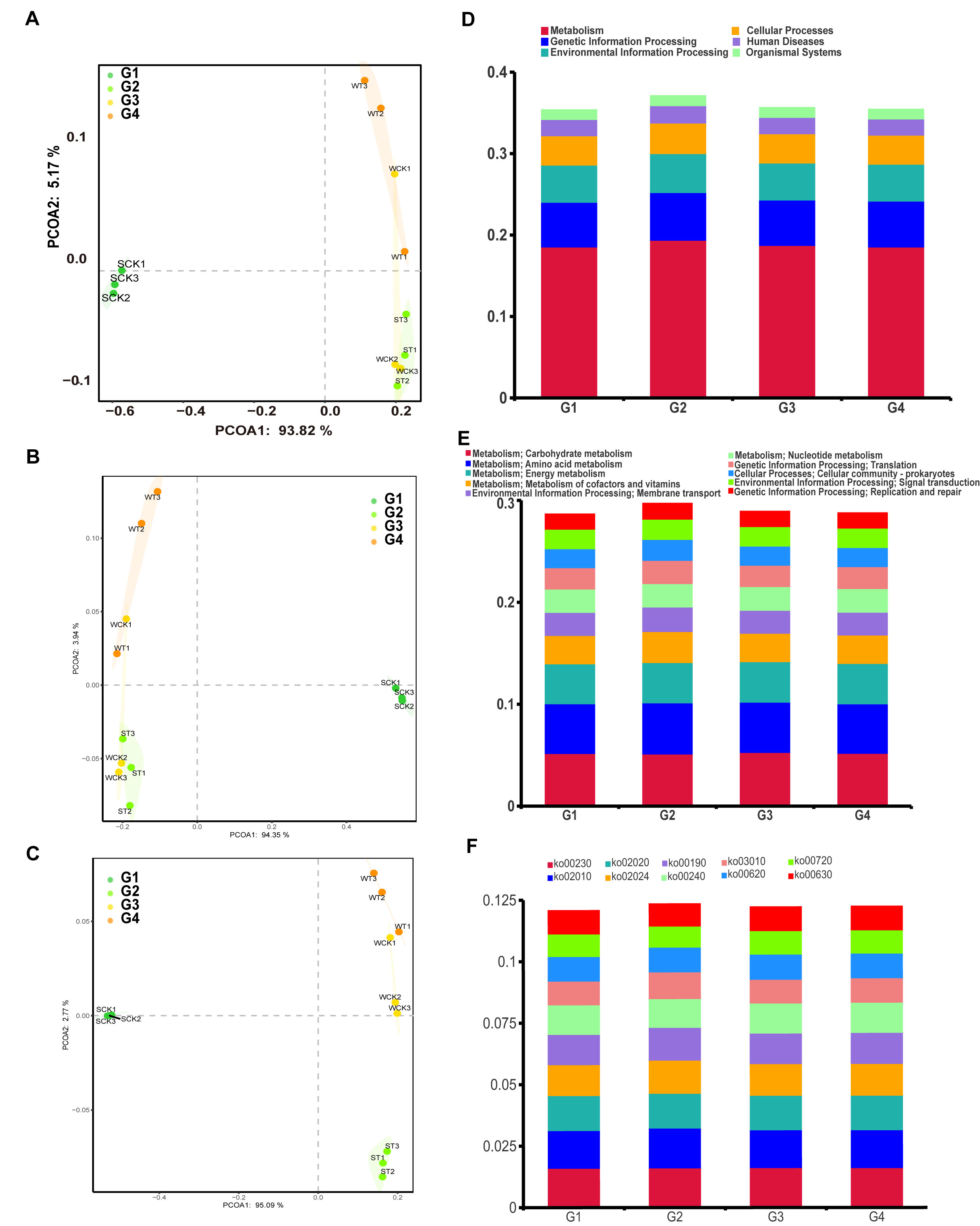

Supplement: Supplementary Figure 4 — KEGG functions among all samples. Principle co-ordinates analysis based on Bray–Curtis distances of microbiota descriptions in different groups at (A) level 1, (B) level 2, and (C) level 3. Relative abundances at (D) level 1, (E) level 2, and (F) level 3. G1, 101-14 treated with NaCl; G2, 101-14 treated with ddH2O; G3, 5BB treated with NaCl; G4, 5BB treated with ddH2O. [file Image_4.JPEG]

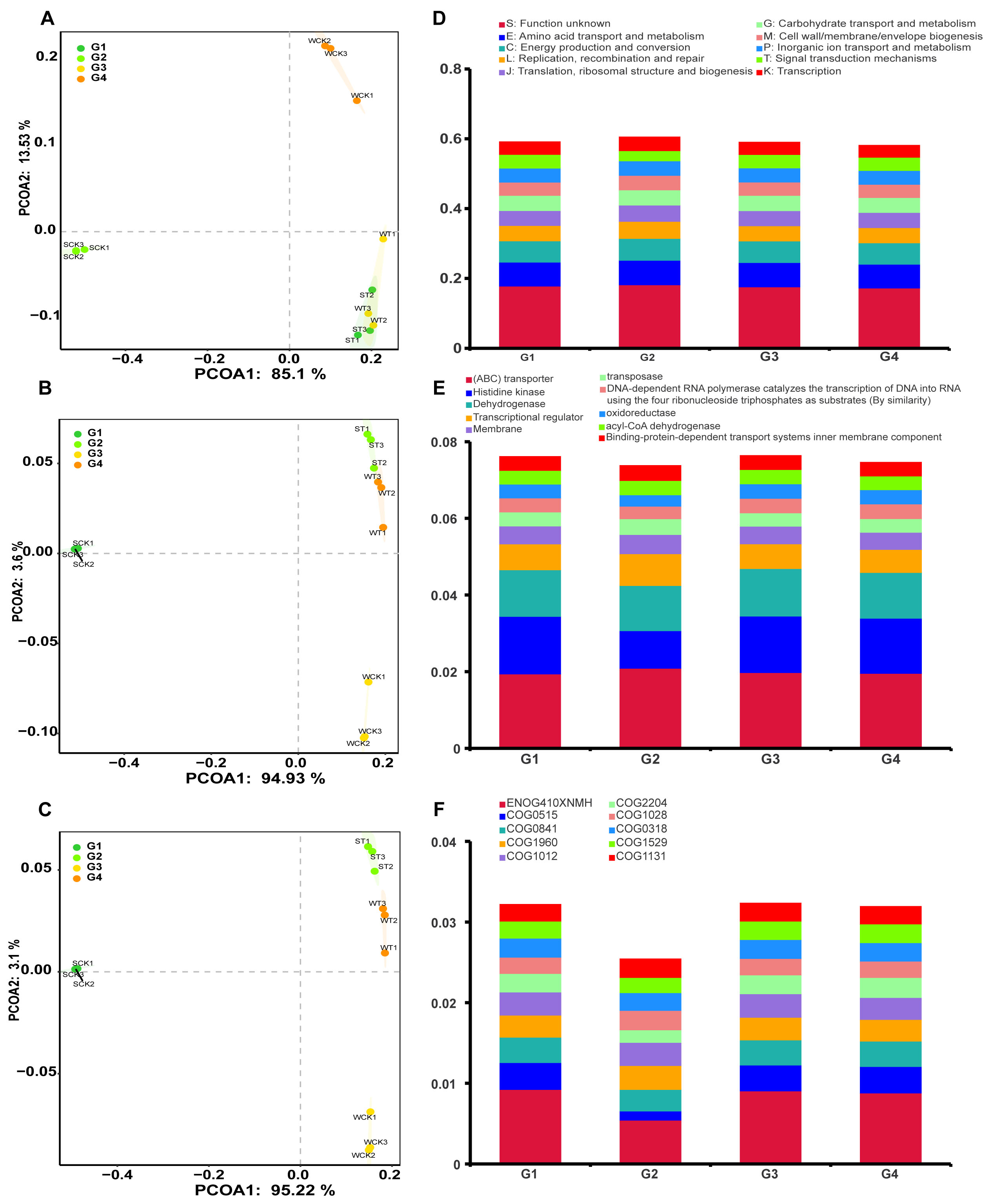

Supplement: Supplementary Figure 5 — Functions identified using the eggNOG database among all samples. Principle co-ordinates analysis based on Bray–Curtis distances of microbiota descriptions in different groups at (A) level 1, (B) level 2, and (C) level 3. Relative abundances at (D) level 1, (E) level 2, and (F) level 3. G1, 101-14 treated with NaCl; G2, 101-14 treated with ddH2O; G3, 5BB treated with NaCl; G4, 5BB treated with ddH2O. [file Image_5.JPEG]

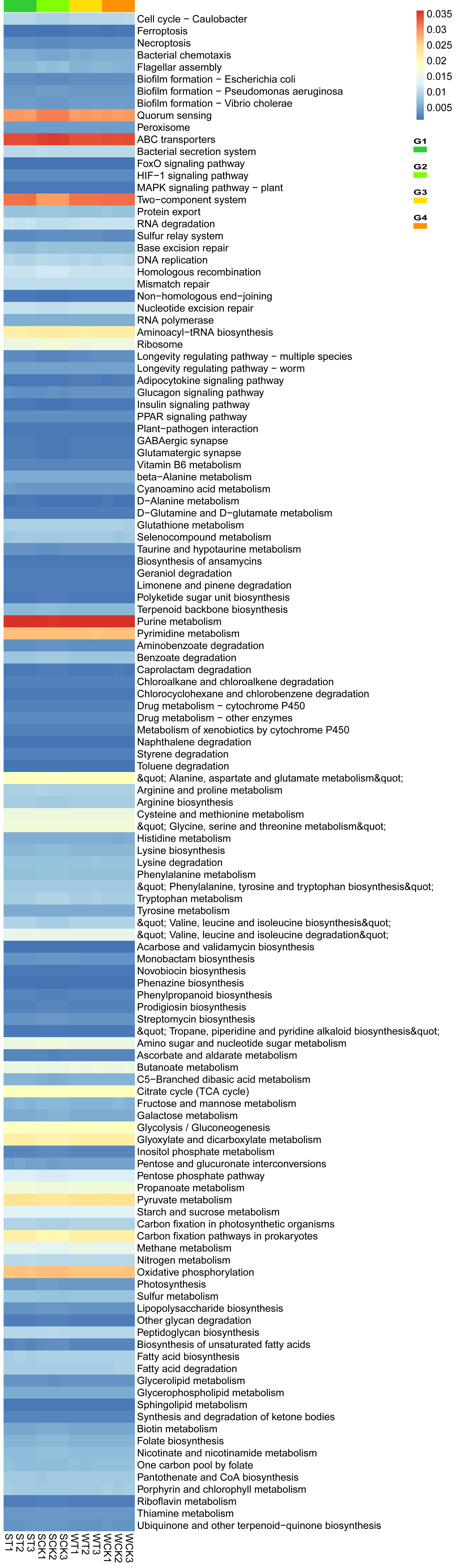

Supplement: Supplementary Figure 6 — Core microbial functions at KEGG level 3 among all samples. G1, 101-14 treated with NaCl; G2, 101-14 treated with ddH2O; G3, 5BB treated with NaCl; G4, 5BB treated with ddH2O. [file Image_6.JPEG]
